# Dual-targeting nanoparticles with excellent gene transfection efficiency for gene therapy of peritoneal metastasis of colorectal cancer

**DOI:** 10.18632/oncotarget.21159

**Published:** 2017-09-22

**Authors:** Ling Li, Rui Deng, Yi Su, Cheng Yang

**Affiliations:** ^1^ The Key Laboratory of Food Colloids and Biotechnology, Ministry of Education, School of Chemical and Material Engineering, Jiangnan University, Wuxi 214122, China; ^2^ Hematology Department and Hematopoietic Stem Cell Transplantation Center, Cheng Du Military General Hospital of PLA, Chengdu 610083, China

**Keywords:** gene therapy, peritoneal metastasis, colorectal cancer, dual-targeting nanoparticle

## Abstract

Colorectal cancer has been one of the most common cancers in the worldwide. Poor patient compliance and serious side effects often associated with conventional therapy (e.g. surgery, radiation, and chemotherapy). Gene therapy may be an alternative strategy. Herein, we developed a dual-targeting nanoparticle with excellent gene transfection efficiency for gene therapy of peritoneal metastasis of colorectal cancer. This nanoparticle can facilitate efficient cellular uptake and promote penetration into nucleus. Meanwhile, this nanoparticle mediated efficient gene transfection in medium with or without serum, which significantly surpassed that of commercial transfection reagents, Lipofectamine 2000 and Lipofectamine 3000. After systemic administration, this nanoparticle loaded with hTRAIL plasmid significantly inhibited peritoneal metastasis of colorectal cancer *in vivo*. In conclusion, this dual-targeting nanoparticle has great potential to be a gene delivery vector for colorectal cancer therapy.

## INTRODUCTION

Nowadays, colorectal cancer has been one of the most common cancers in the worldwide [1, 2]. The primary metastasis mode of colorectal cancer is that dissemination occurs *via* tumor cells shedding into the peritoneal cavity, survival and growth within ascites, and re-adhesion and proliferation within the abdomen. Currently, conventional therapies, such as surgery, radiation, and chemotherapy, have been commonly applied for management of colorectal cancer. However, poor patient compliance and serious side effects largely limited their wide applications [3, 4]. Gene therapy, which holds great promise in treating inherited and acquired diseases, may be an alternative strategy [5, 6].

Tumor necrosis factor-related apoptosis-inducing ligand (TRAIL) has been widely used as a cancer therapeutic. TRAIL triggers apoptosis through interaction with the death receptors DR4 and DR5 [7]. It has been found that DR4 and DR5 were highly overexpressed in the colorectal cancer samples. Besides, some reports had demonstrated that human colon carcinoma cells, such as HCT 116 and SW 480 (human colon cancer cell lines), are sensitive to the apoptosis mediated by TRAIL [8]. Therefore, TRAIL may be a potential therapeutic for the treatment of colorectal cancer.

**Figure 1:**
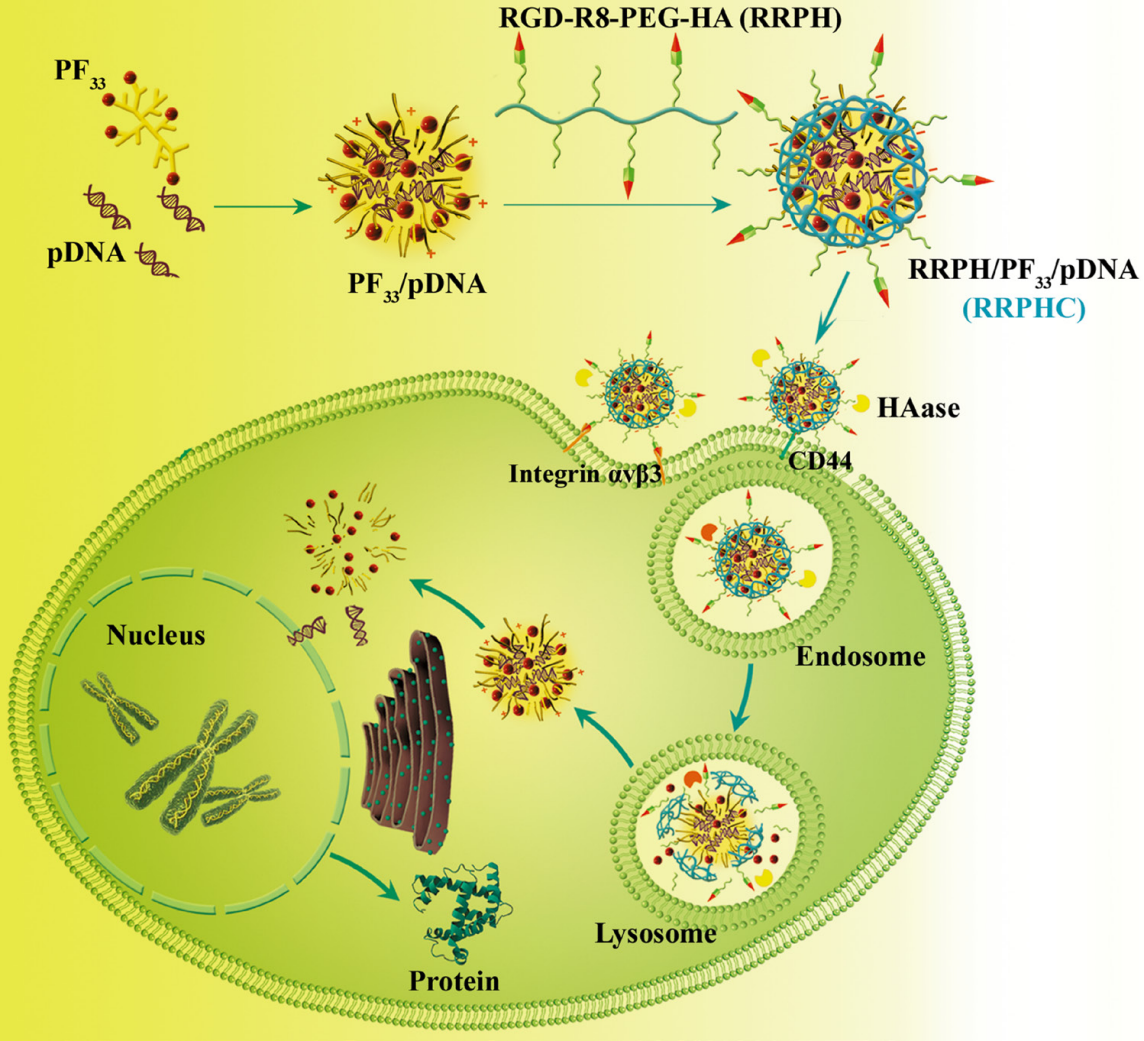
Schematic illustration of the preparation and dual tumor-targeting of RRPHC ternary nanoparticles

In addition to therapeutic genes, gene carriers are also critical for gene therapy. In this context, we construct a ternary nanoparticle (RRPH/PF_33_/pDNA, RRPHC) with dual active targeting capability for *in vitro* and *in vivo* gene delivery (Figure 1). The system consists of a binary nanoparticle core (PF_33_/pDNA) with excellent gene transfection efficiency and a negatively charged shell (RGD-R8-PEG-HA, RRPH) with dual-targeting capability. RRPH was hyaluronan (HA) polymers grafted with PEG chains, which were further conjugated with RGD-R8 peptide. RRPH polymer can specifically interact with CD44 receptors overexpressed on the surface of many types of tumors [8–10]. Meanwhile, RRPH polymer possessed both specific targeting to integrin α_v_β_3_ receptor and high penetrating ability attributed to the RGD-R8 peptide moiety [11]. As we know, integrin α_v_β_3_ receptors were overexpressed on tumor neovasculature and many types of tumor (such as melanoma, breast cancer, colon cancers etc) [12, 13].

In this work, we designed a dual-targeting nanoparticle for *in vitro* and *in vivo* gene therapy of colorectal cancer. Cellular uptake, dual active targeting, intracellular distribution, and gene transfection were carefully evaluated *in vitro*. Finally, the anti-tumor efficacies were further evaluated in peritoneal metastasis model of human colon carcinoma cells *in vivo*.

## RESULTS AND DISCUSSION

### Characterization of PF_33_/pDNA and RRPHC/pDNA nanoparticles

**Figure 2:**
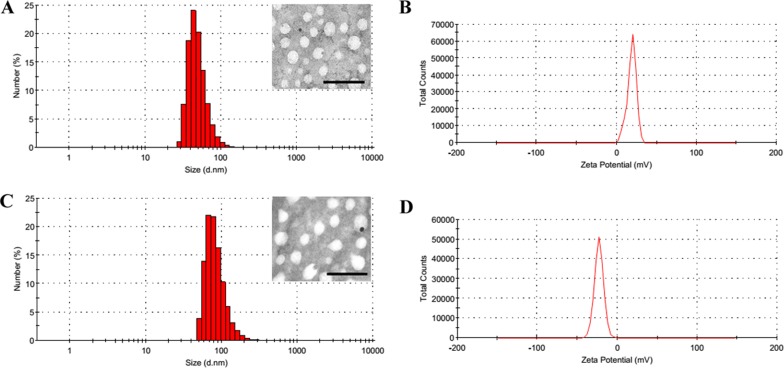
Characterization of PF_33_/pDNA and RRPHC/pDNA nanoparticles (**A**, **B**) Hydrodynamic size and zeta potential of PF_33_/pDNA nanoparticles measured by DLS. Inset: TEM image of PF_33_/pDNA nanoparticles, scale bar indicates 100 nm. (**C**, **D**) Hydrodynamic size and zeta potential of RRPHC/pDNA nanoparticles measured by DLS. Inset: TEM image of RRPHC/pDNA nanoparticles, scale bar indicates 100 nm.

In the past decades, fluorine was widely applied to medicinal chemistry and greatly promoted the drug development. Highly fluorinated compounds have unique properties, such as both hydrophobic and lipophobic, and high phase-separation tendency [14, 15]. According to the previous study [16, 17], we synthesized a series of cationic fluorinated polymers (PFs) and found PF_33_ (the percentage of fluorinated groups modified was ~33%) showed excellent transfection efficacy. The PF_33_/pDNA nanoparticles exhibited a hydrodynamic diameter of 81.3 nm and a moderate positive zeta potential of +18.8 mV (Figure 2A and 2B). After coated with RRPH polymer, the hydrodynamic size showed moderate increase to 126.7 nm, while the surface charge became negative (−23.0 mV) (Figure 1C and 1D). Transmission electron microscopy (TEM) measurement confirmed that both nanoparticles before and after coating exhibited compact and spheroid morphology (Figure 2A and 2C).

**Figure 3:**
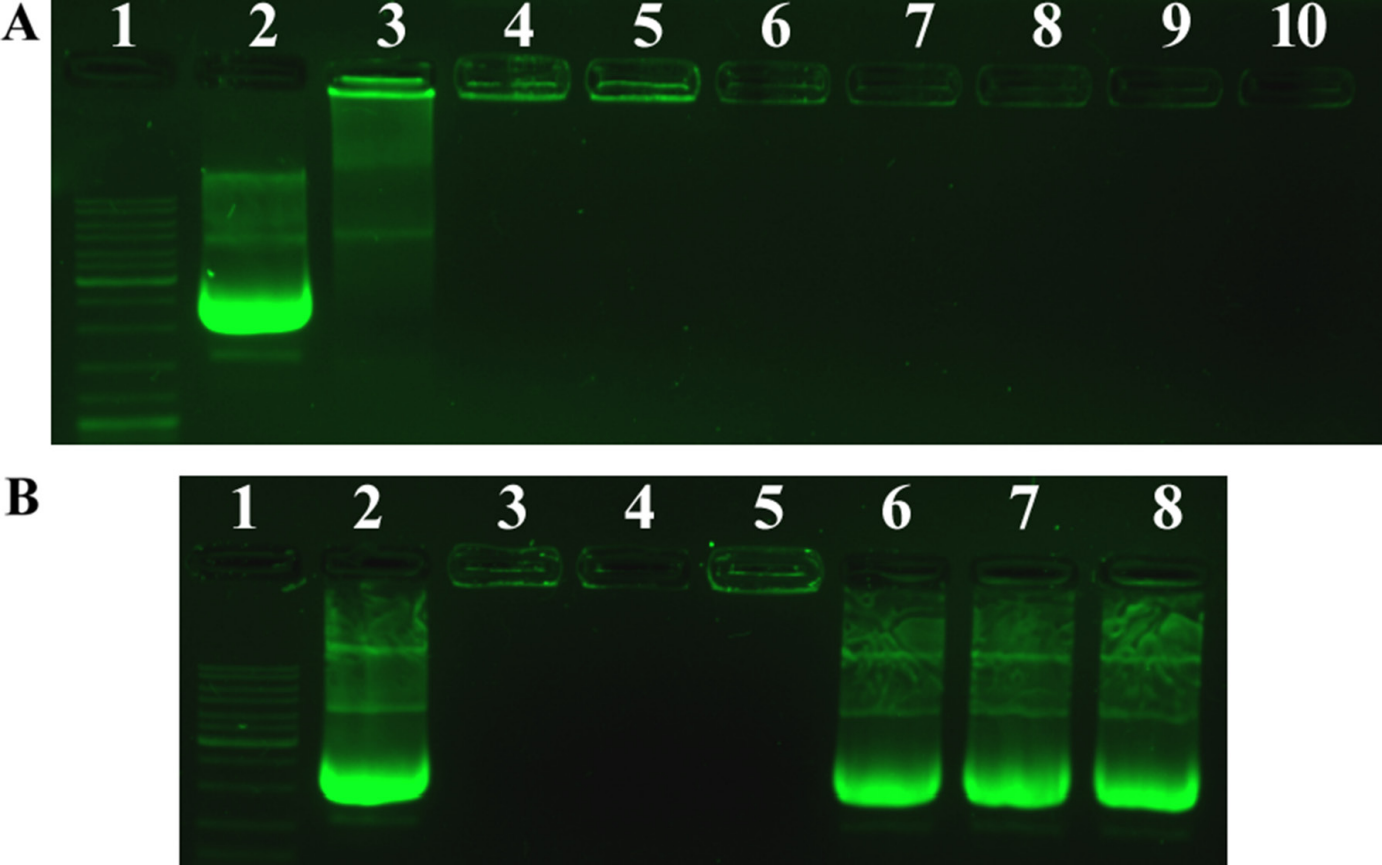
Agarose gel electrophoresis of PF_33_/pDNA complexes, HAC/pDNA complexes and RRPHC/pDNA nanoparticles (**A**) Lane 1, DNA ladder; lane 2, naked pDNA; lane 3–8, PF_33_/pDNA at mass ratios of 0.5:1, 1:1, 2:1, 4:1, 8:1, 10:1; lane 9, HAC/pDNA; lane 10, RRPHC/pDNA. (**B**) Lane 1, DNA ladder; lane 2, naked pDNA; lane 3, PF_33_/pDNA at mass ratio of 10:1; lane 4, HAC/pDNA; lane 5, RRPHC/pDNA; lane 6, PF_33_/pDNA after dissociation; lane 7, HAC/pDNA after dissociation; lane 8, RRPHC/pDNA after dissociation.

Next, we performed the agarose gel retardation assay to assess the condensation ability of PF_33_ polymer. As shown in Figure 3A, the DNA mobility was totally retarded at mass ratio above 1:1 (PF_33_: pDNA). The DNA signal was observed in the well and there was no DNA band appeared in the gel. After coated with HA or RRPH polymer, no DNA detachment was observed. Besides, in order to further confirm the existence of pDNA in the nanoparticles, we dissociated the above nanoparticles with Triton X-100 and heparin. As shown in Figure 2B, the nanoparticles without disruption were located in the lane, while after dissociation, the pDNA recovered from the nanoparticles showed an obvious band in the agarose gel, further confirming that the formed nanoparticles contained pDNA.

### Cellular uptake analysis

**Figure 4:**
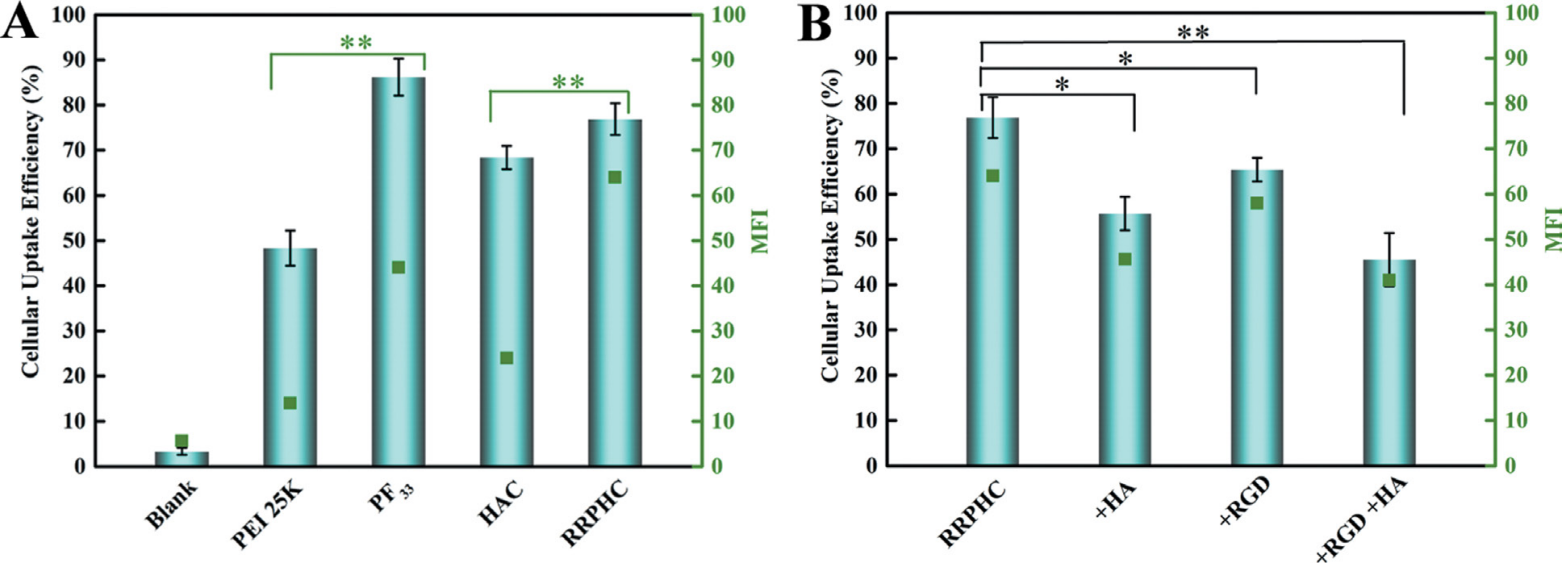
Cellular uptake of the prepared nanoparticles in SW 480 cells (**A**) Quantitative analysis of the cellular uptake efficiency of different nanoparticles measured by flow cytometry. (**B**) Quantitative analysis of the cellular uptake efficiency of RRPHC/pDNA nanoparticles after addition of excess HA or (and) RGD peptide to saturate CD44 or (and) integrin α_v_β_3_ receptors. *p = 0.0245 and **p = 0.0037.

We then measured the cellular uptake efficiency of the above nanoparticles in SW480 cells by flow cytometry. The polyplexes of PEI 25K/pDNA were performed as controls. As shown in Figure 4A, both PF_33_/pDNA and RRPHC/pDNA nanoparticles exhibited comparable efficient cellular uptake in SW480 cells (~80%). Meanwhile, we also observed the negatively charged HAC/pDNA complexes exhibited higher cellular uptake efficiency (~65%) than PEI 25K/pDNA (~45%). To further evaluate whether the effective cellular uptake of RRPHC/pDNA complexes in SW480 cells was associated with CD44 and integrin α_v_β_3_ receptors, the CD44 or (and) integrin α_v_β_3_ receptors on the surface of SW480 cells were pre-blocked. As shown in Figure 4B, after addition of excess HA or RGD peptide, the cellular uptake efficacy of RRPHC/pDNA complexes significantly decreased, validating the enhanced cellular uptake of RRPHC/pDNA complexes was largely due to the dual receptor-mediated endocytosis.

### Intracellular distribution

**Figure 5:**
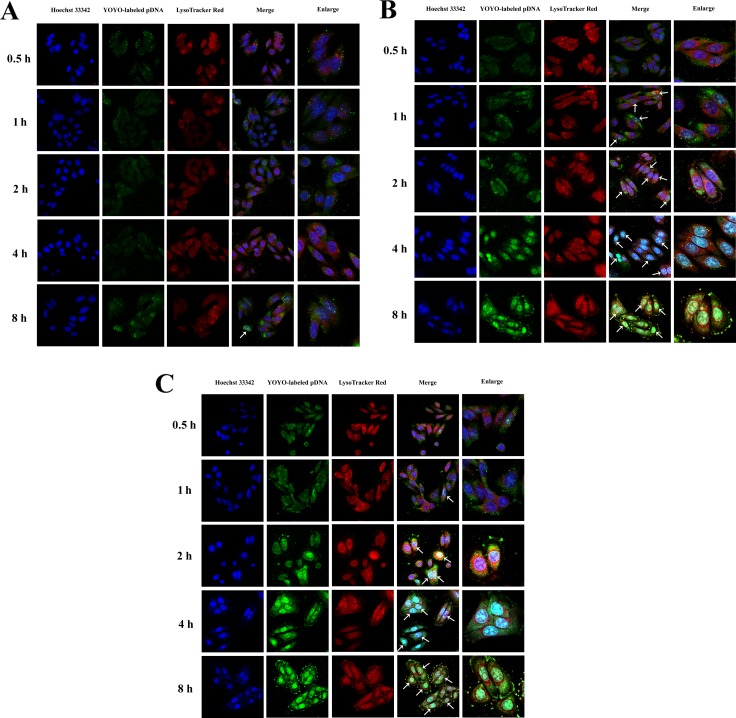
Intracellular distribution of PEI 25K/pDNA (**A**), PF_33_/pDNA (**B**) and RRPHC/pDNA (**C**) nanoparticles in SW 480 cells at 0.5, 1, 2, 4 and 8 h, respectively. pDNA was labeled with YOYO-1, the endosomes and lysosomes were stained with Lyso-Tracker Red, while the nuclei were stained with Hoechst 33342. The arrows indicate co-localization of YOYO-1 labeled pDNA and the nuclei.

Since efficient penetration to the nuclei is needed to fully realize gene transfection of pDNA, we further evaluated the intracellular distribution of PF_33_/pDNA and RRPHC/pDNA nanoparticles in SW480 cells. The polyplexes of PEI 25K were applied as controls. As presented in Figure 5, the intracellular distribution of nanoparticles was presented in a time-dependent manner. In the PEI 25K/pDNA treated group, the pDNA began to accumulate in the nuclei after incubation for 8h, while the pDNA of the PF_33_/pDNA nanoparticles were already associated with the nuclei in the first 2 h. Almost all the nuclei were completely overlapped with the green signal of pDNA within 4 h of incubation. The situation of RRPHC/pDNA nanoparticles was similar with PF_33_/pDNA nanoparticles.

### *In vitro* gene transfection

**Figure 6:**
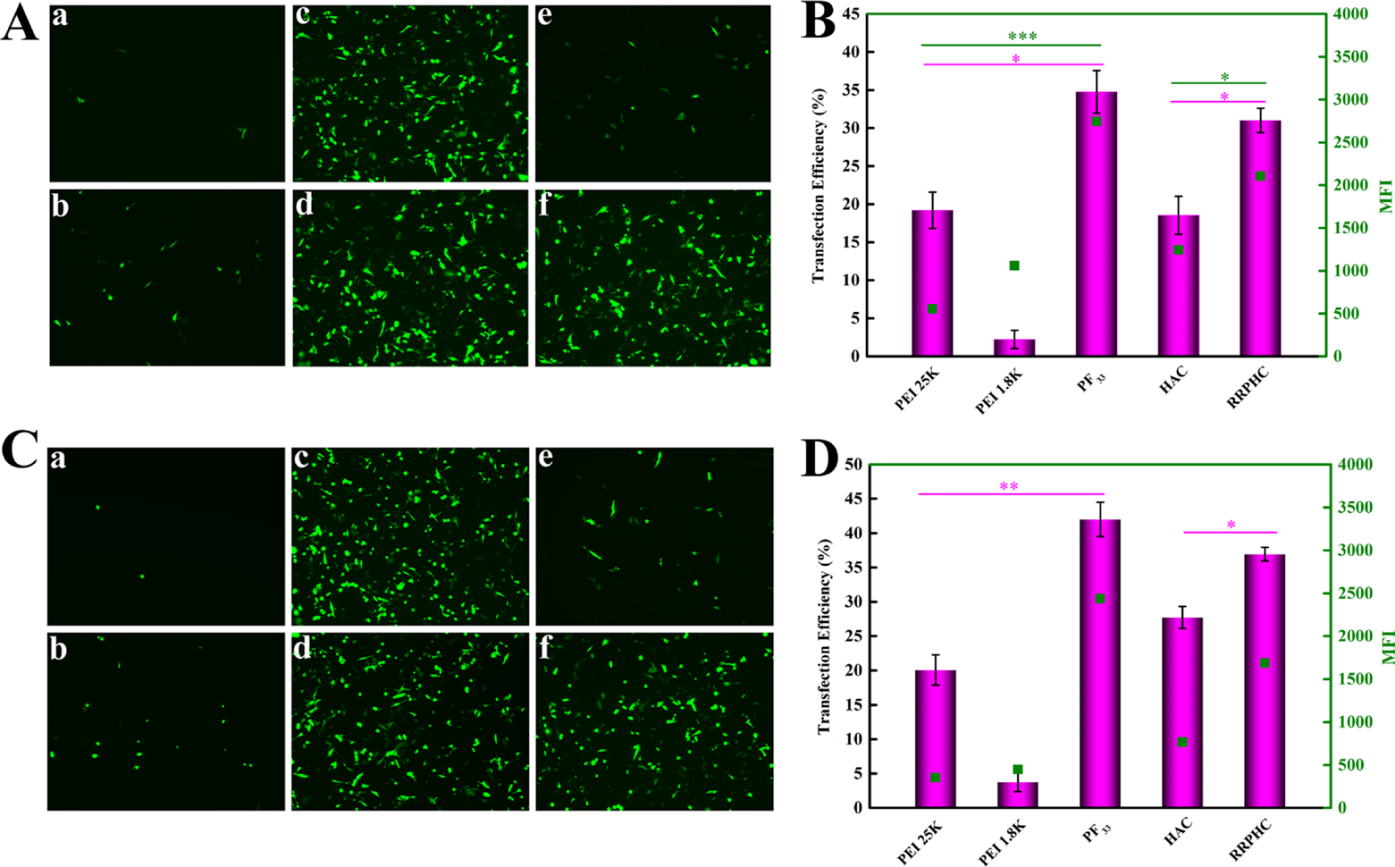
*In vitro* gene transfection efficacies of different nanoparticles in serum-free medium in SW 480 cells at 24 h (**A**, **B**) and 48 h (**C**, **D**). (A, C) PEI 1.8K/pGFP (a), PEI 25K/pGFP (b), PF_33_/pGFP at mass ratio of 5:1 (c), 10:1(d), HAC/pGFP (e) and RRPHC/pGFP (f). (B, D) Quantitative analysis of transfection efficiency by flow cytometry. *p = 0.0316, **p = 0.00524 and ***p = 0.0003.

In the next study, we focused on evaluating the *in vitro* gene transfection efficiency of PF_33_/pDNA and RRPHC/pDNA nanoparticles in SW480 cells. The polyplexes of PEI 25K and PEI 1.8K were performed as controls. The gene transfection in serum-free medium was firstly conducted. As shown in Figure 6A and 6B, the PF_33_/pDNA nanoparticles mediated efficient gene transfection efficiency (~ 35%) in SW 480 cells at 24 h, much higher than that of PEI 25K (< 20%) and PEI 1.8K (< 5%). RRPHC/pDNA nanoparticles induced comparable gene transfection efficiency with PF_33_/pDNA nanoparticles, much higher than that of HAC/pDNA nanoparticles (< 20%). The gene transfection results at 48 h were similar with that of 24 h, except the gene transfection efficiency of both PF_33_/pDNA nanoparticles and RRPHC/pDNA nanoparticles increased to some extent (Figure 6C and 6D).

**Figure 7:**
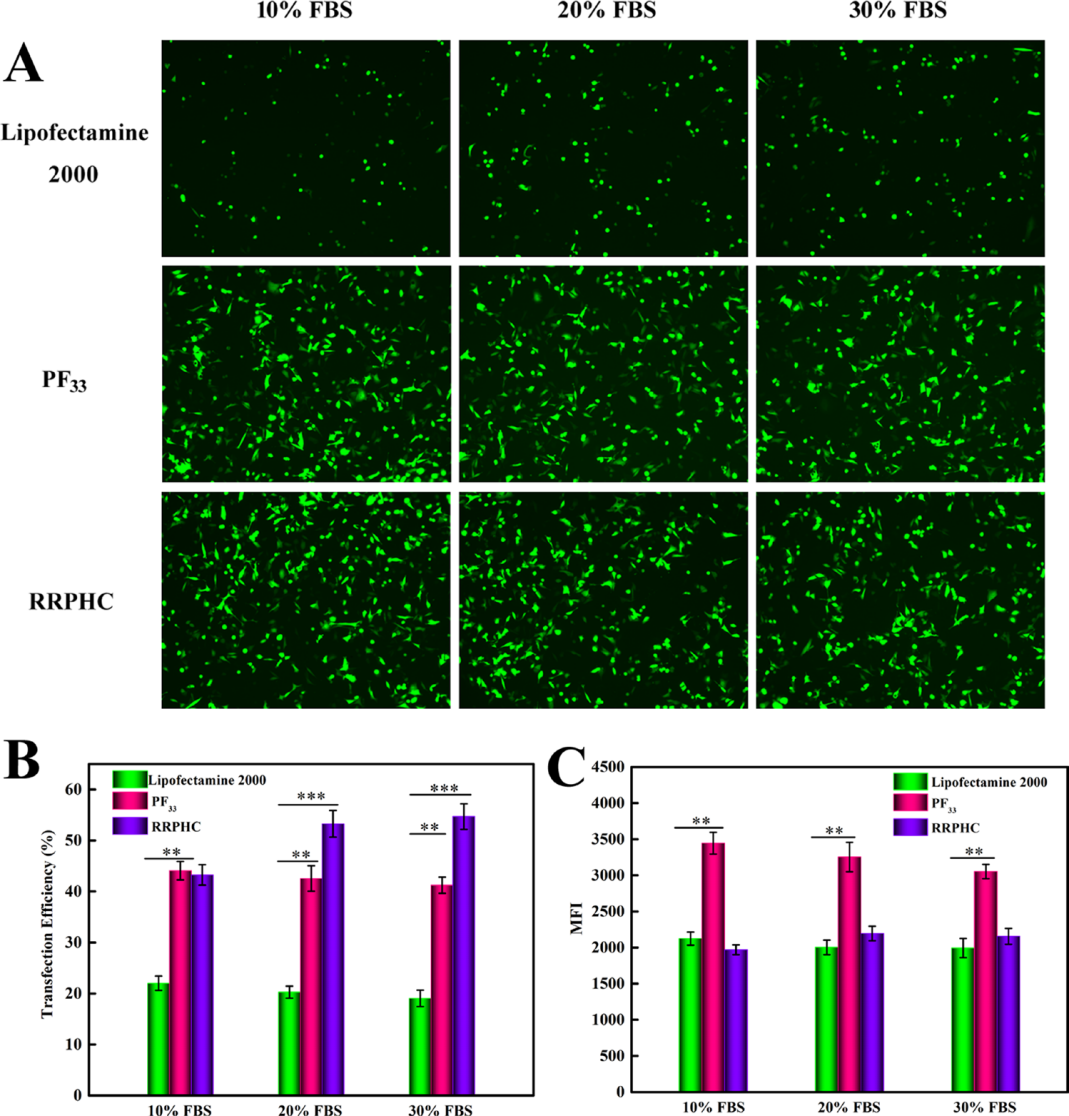
Comparison of the transfection efficiency of PF_33_/pGFP (PF_33_), RRPHC/pGFP (RRPHC) and Lipofectamine 2000/pGFP (Lipofectamine 2000) in medium containing 10%~30% serum in SW 480 cell (**A**) Images taken by fluorescence microscope. (**B**, **C**) Quantitative analysis of positive GFP cells (%) and Mean Fluorescence Intensity (MFI) by flow cytometry.

**Figure 8:**
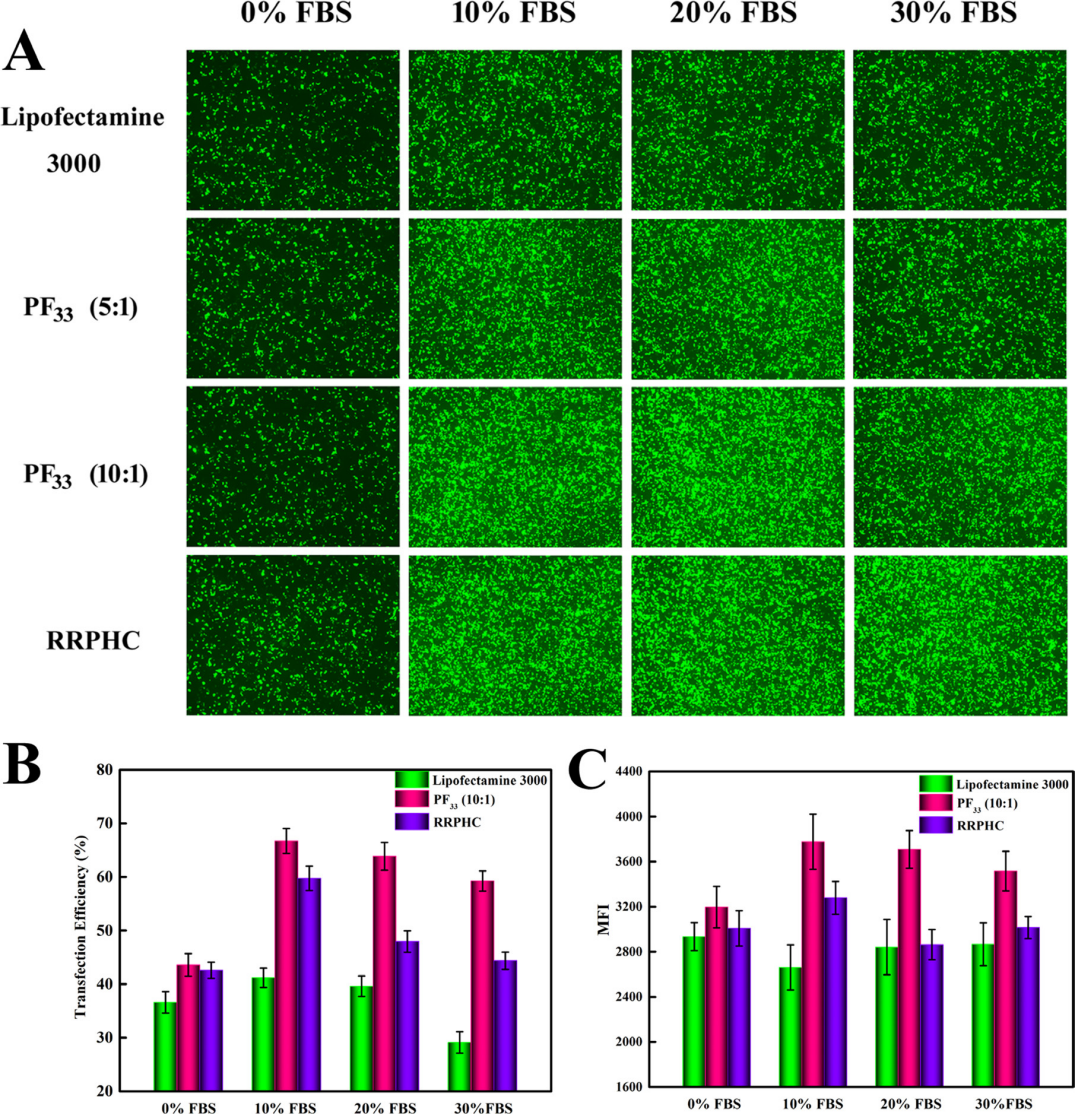
Comparison of the transfection efficiency of PF_33_/pGFP (PF_33_), RRPHC/pGFP (RRPHC) and Lipofectamine 3000/pGFP (Lipofectamine 3000) in medium containing 0%~30% serum in SW 480 cell (**A**) Images taken by fluorescence microscope. (**B**, **C**) Quantitative analysis of positive GFP cells (%) and Mean Fluorescence Intensity (MFI) by flow cytometry.

Next, we evaluated the transfection efficiency of the above nanoparticles in medium containing serum. The lipoplexes of Lipofectamine 2000 was used as control. As presented in Figure 7, both PF_33_/pDNA and RRPHC/pDNA nanoparticles remained their effective gene transfection ability in medium containing 10~30% serum (> 40%), much higher than that of Lipofectamine 2000 (~ 20%). Besides, we noted that many cells became round-shaped after transfection with the lipoplexes of Lipofectamine 2000, implying cytotoxicity of Lipofectamine 2000. Moreover, the gene transfection efficacy of PF_33_/pDNA and RRPHC/pDNA nanoparticles was further compared with the lipoplexes of Lipofectamine 3000, which is a commercial updated transfection reagent of Lipofectamine 2000. Both PF_33_/pDNA and RRPHC/pDNA nanoparticles exhibited much higher gene transfection efficiency than that of Lipofectamine 3000 in medium containing 10~30% serum in SW 480 cells (Figure 8).

### Apoptosis-inducing effect evaluation

**Figure 9:**
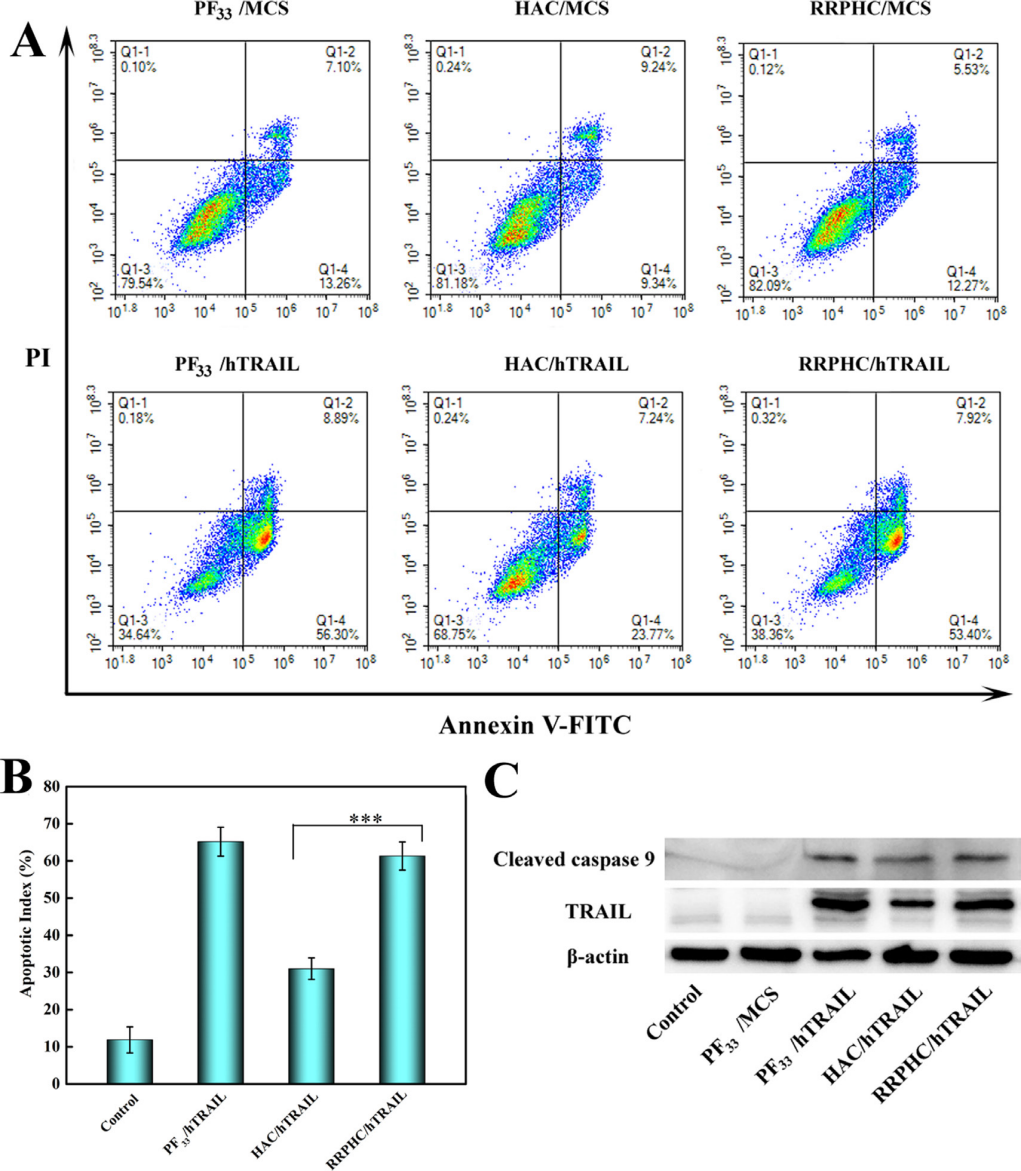
(**A**, **B**) *In vitro* apoptosis-inducing effect in SW 480 cells after transfection with different formulations loaded with hTRAIL plasmid at 24 h. Early apoptotic cells are shown in the lower right quadrant (Q2–4), and late apoptotic cells are shown in the upper right quadrant (Q2–2). (**C**) Analysis the expression levels of TRAIL and cleaved caspase 9 protein in SW 480 cells after transfection with different formulations. ***p = 0.0006.

Inspired by the above great results, we further evaluated the apoptosis-inducing effect of the nanoparticles loaded with hTRAIL plasmid. As shown in Figure 9A and 9B, PF_33_/hTRAIL nanoparticles induced ~65% of total apoptosis at 24 h in SW 480 cells. The RRPHC/hTRAIL nanparticles led to comparable apoptosis effect (~60%) with PF_33_/hTRAIL nanoparticles, much higher than that of HAC/hTRAIL nanoparticles (~30%). It's worth noting that all nanoparticles loading with MCS plasmid (control vector without target sequence) induced minimal apoptosis-inducing effect. Furthermore, western blotting analysis was performed to examine the expression of TRAIL protein after transfection. As presented in Figure 9C, both PF_33_/hTRAIL and RRPHC/hTRAIL nanoparticles significantly promoted the expression of TRAIL protein in SW 480 cells, more than that after HAC/hTRAIL nanoparticles treatment. These results were well correlated with the previous gene transfection efficiency. Meanwhile, the expression of the cleaved caspase 9, the pro-apoptotic protein, also increased in SW 480 cells after treated with PF_33_/hTRAIL and RRPHC/hTRAIL nanoparticles.

### *In vivo* anti-tumor activity evaluation

**Figure 10:**
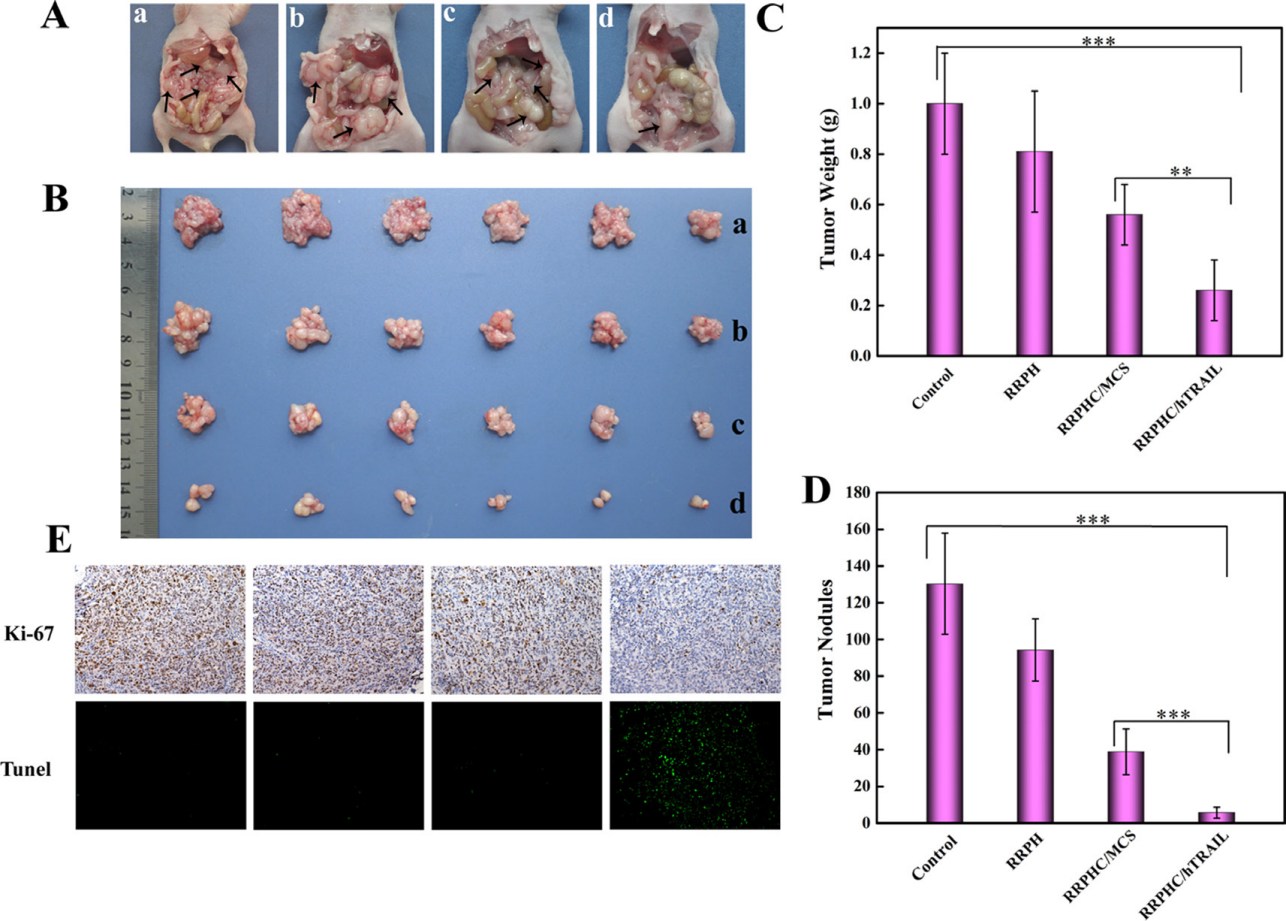
*In vivo* anti-tumor potency evaluation (**A**, **B**) Representative photographs of abdominal metastatic nodes (arrows) in each treatment group. PBS (a), RRPH polymer (b), RRPHC/MCS (c), and RRPHC/hTRAIL (d). (**C**, **D**) Tumor weights and tumor nodules after treatment with different formulations. (**E**) IHC analyses of the expression of Ki-67 and TUNEL in tumor tissues of different treatment groups. **p = 0.0052, ***p = 0.0005.

Finally, the therapeutic anti-tumor potential of RRPHC/hTRAIL nanoparticles was further evaluated in peritoneal metastasis model of SW 480 cells *in vivo*. As shown in Figure 10. RRPHC/hTRAIL nanoparticles significantly inhibited the growth of peritoneal metastasis tumor. At the end point of treatment, the peritoneal organs including omentum surface, colon bag, below the diaphragm, stomach and so on of the mice treated with PBS (phosphate buffer saline) or RRPH polymer were almost completely occupied by metastasized colonies, while only a few metastasized colonies could be observed after treatment with RRPHC/hTRAIL nanoparticles (Figure 10A). Additionally, we also evaluated the proliferation and apoptosis of tumor cells in tumor tissues after treatment. RRPHC/hTRAIL was more effective in preventing tumor cell proliferation with less Ki-67-positive tumor cells in tumor tissues. Meanwhile, the strongest apoptosis effect in tumor cells was also observed after RRPHC/hTRAIL treatment. In sharp contrast, the control groups including PBS, RRPH polymers and RRPHC/MCS didn't significantly affect proliferation and apoptosis of tumor cells (Figure 10E). Additionally, RRPHC/hTRAIL treatment did not cause any toxicity to normal tissues and organs according to the histological analysis (Data not shown).

## MATERIALS AND METHODS

### Materials

R8-RGD peptides with a terminal cysteine modification [Cys-RRRRRRRR-c(RGDfK)] were purchased from Chinapeptides Co. Ltd. (Shanghai, China). PEI 25K was purchased from Sigma-aldrich and PEI 1.8K was purchased from Alfa Aesar. YOYO-1, Lyso-Tracker red, Hoechst 33342, Lipofectamine 2000 and Lipofectamine 3000 were purchased from Invitrogen (USA). pUNO1-hTRAILa (hTRAIL) and pUNO1-MCS (MCS) plasmid were obtained from *In vivo* Gen (San Diego, CA, USA) and purified with QIAGEN Plasmid Mega Kit (Qiagen GmbH, Hilden, Germany). Annexin V-FITC /PI apoptosis detection kit was purchased from Nanjing KeyGen Biotech. Co., Ltd (Nanjing,China). Antibodies used for western blotting were purchased from Cell Signaling Technology (CST, Beverly, MA, USA).

SW 480 cell line was obtained from American Type Culture Collection (ATCC, USA) and cultured in DMEM with 10% fetal bovine serum (Gibco), 100 units/mL penicillin G sodium and 100 μg/mL streptomycin sulfate and maintained at 37 °C in a humidified and 5% CO2 incubator.

### Preparation and characterization of PF_33_/pDNA and RRPHC/pDNA nanoparticles

PF_33_ and RRPH polymer was synthesized according to previous reports [18, 19]. The PF_33_/pDNA nanoparticles were obtained by briefly mixing 2 μg of pDNA with 20 μg of PF_33_ polymer and incubated at room temperature for 30 min. To prepare RRPHC/pDNA or HAC/pDNA nanoparticles, 60 μg of RRPH polymer or HA polymer was added to the above binary nanoparticles and further incubated for another 20 min, respectively. The hydrodynamic size and zeta potential of the prepared nanparticles were measured by a dynamic light scattering (DLS) instrument (Malvern Zetasizer Nano-S, Malvern Inc., UK), while the morphologies were further examined by TEM (JEOL JEM-100CX, Japan).

The condensation ability of the prepared nanoparticles was assessed by gel retardation assays. pDNA was incubated with PF_33_ polymer in distilled water to form the PF_33_/pDNA nanoparticles for 30 min with different mass ratios (0.5, 1, 2, 4, 8, and 10). Then these nanoparticles were loaded onto 1% (w/v) agarose gel (pre-mixed with Gelview) and electrophoresed at 120 V for 30 min. DNA retardation was recorded by a UV illuminator (Bio-Rad ChemiDoc XRS+, USA). To release the pDNA from the prepared nanoparticles, 5 μl of Triton-X 100 was added to the solution and incubated for 10 min to dissociate the nanoparticles. Then heparin sulfate was added to mixture and incubated for another 15 min and subjected to agarose gel electrophoresis [20, 21].

### *In vitro* cellular uptake

pDNA was pre-labeled with YOYO-1 (nucleic acid dye). SW 480 cells were plated into 6-well plate (3 × 10^5^ cells/well) and cultured for 30 h. Then the nanoparticles loaded with 2 μg of YOYO-1 labeled pDNA were added to each well and further incubated for another 2 h at 37°C. After incubation, the cells were rinsed with PBS and harvested for the following flow cytometry analysis. In the competitive receptor study, cells were pre-incubated with excess free HA (final concentration, 10 mg/mL) or (and) RGD peptide (final concentration, 25 mM) for 1 h before addition of the nanoparticles.

### Intracellular distribution

SW 480 cells were seeded into 6-well plate (3 × 10^5^ cells/well) over glass cover slips and incubated for 30 h. Then the nanoparticles loaded with 2 μg of YOYO-1 labeled pDNA were added to the medium. Lysosomes and endosomes were stained with LysoTracker Red for 1 h before harvesting cells. At determined time point (0.5, 1, 2, 4, 8 h), cells were rinsed and incubated with Hoechst 33342 for 10 min. Subsequently, cells were then washed with PBS, fixed with 4% paraformaldehyde and analyzed with confocal laser scanning microscopy (CLSM, ZEISS, LSM 880, Germany).

### *In vitro* gene transfection

To evaluate the transfection efficiency of the prepared nanoparticles in SW 480 cells, pGFP (Green Fluorescent Protein plasmid) was used as reporter gene. SW 480 cells were seeded into 6-well plates (3.0 × 10^5^ cells/well) and cultured for 30 h before transfection. The medium was replaced with fresh serum-free medium or medium containing 10~30% FBS. The nanoparticles loaded with 2 μg of pGFP were added to each well and incubated for another 4 h. After incubation, the medium was replaced with fresh medium containing 10% serum and further incubated for additional 24 or 48 h. PEI 25K and PEI 1.8K, Lipofectamine 2000 and Lipofectamine 3000 was conducted at their optimal conditions. Finally, the cells were rinsed with PBS and subjected to the inverted fluorescence microscope (Olympus, Japan) and the flow cytometry (Calibur, BD, USA).

### *In vitro* apoptosis effect ananlysis

The apoptosis of SW 480 cells after treatment with the nanoparticles loaded with hTRAIL gene was quantitatively measured by Annexin V/PI apoptosis detection kit. Briefly, SW 480 cells were seeded into 6-well plates (3.0 × 10^5^ cells/well) and incubated for 30 h before transfection. Then the cells were transfected with the nanoparticles loaded with 2 μg of hTRAIL or MCS plasmid for 4 h, respectively. Subsequently, the medium was replaced with fresh medium containing 10% serum and further incubated for another 24 h. After incubation, cells were trypsinized, washed with cold PBS, and resuspended in binding buffer. 5 μL of Annexin-V-FITC and PI was added to the cells and incubated for 10 min.

In western blotting analysis, total protein of the cells treated with different nanoparticles was extracted. ~30 μg of total protein was separated by 15% sodium dodecyl sulfate-polyacrylamide gel electrophoresis (SDS-PAGE) and transferred to PVDF membranes. The PVDF membranes were blocked in 5% non-fat milk for 2 h and incubated with antibodies against TRAIL and cleaved caspase 9 at 4 °C overnight, followed by washing with Tris buffered saline solution with the detergent Tween-20 (TBST). Subsequently, the washed PVDF membranes were incubated with HRP-conjugated secondary antibodies in 5% non-fat milk for 45 min at 37 °C. After incubation, the membranes were then washed with TBST and the protein bands were visualized using a chemiluminescence (ECL) detection system.

### *In vivo* anti-tumor efficacy

The female BALB/c nude mice (6–8 week) were received from the Vital Laboratory Animal Center (Beijing, China). All animals were treated in accordance with the Guide for Care and Use of Laboratory Animals, approved by the Ethics Committee of Cheng Du Military General Hospital of PLA. The peritoneal metastasis model of SW 480 cells was established by intraperitoneal injection of SW 480 cells (1×10^7^ cells for each mouse). Ten days after inoculation, mice were randomly allocated into four groups and intraperitoneally administered with PBS, RRPH (150 μg), RRPHC/MCS and RRPHC/hTRAIL once every three days, respectively. The dosage of pDNA of each injection was 5 μg per mouse. All mice were euthanized at 30 days after tumor challenge. The metastatic colonies were counted and the tumors were weighed. The tumor tissues were then fixed in 4% paraformaldehyde for further immunohistochemical (IHC) analysis.

### Statistical analysis

The data collected were presented as mean ± standard deviation (S.D.). Statistic analysis was performed by one-way ANOVA. Significant differences between groups were indicated by *p < 0.05, **p < 0.01 and ***p < 0.001, respectively.

## References

[1] Miller KD, Siegel RL, Lin CC, Mariotto AB, Kramer JL, Rowland JH, Stein KD, Alteri R, Jemal A (2016). Cancer treatment and survivorship statistics. CA Cancer J Clin.

[2] Ferlay J, Soerjomataram I, Dikshit R, Eser S, Mathers C, Rebelo M, Parkin DM, Forman D, Bray F (2015). Cancer incidence and mortality worldwide: Sources, methods and major patterns in GLOBOCAN 2012. International Journal of Cancer.

[3] Ubel PA, Abernethy AP, Zafar SY (2013). Full disclosure--out-of-pocket costs as side effects. New England Journal of Medicine.

[4] Mayer C, Popanda O, Greve B, Fritz E, Illig T, Eckardt-Schupp F, Gomolka M, Benner A, Schmezer P (2011). A radiation-induced gene expression signature as a tool to predict acute radiotherapy-induced adverse side effects. Cancer Letters.

[5] Li L, Wei Y, Gong C (2015). Polymeric Nanocarriers for Non-Viral Gene Delivery. Journal of Biomedical Nanotechnology.

[6] Centlivre M, Legrand N, Klamer S, Ying PL, Eije KJV, Bohne M, Rijnstra ES, Weijer K, Blom B, Voermans C (2013). Preclinical In Vivo Evaluation of the Safety of a Multi-shRNA-based Gene Therapy Against HIV-1. Mol Ther Nucleic Acids.

[7] Di PR Zauli G (2004). Emerging non-apoptotic functions of tumor necrosis factor-related apoptosis-inducing ligand (TRAIL)/Apo2L. Journal of Cellular Physiology.

[8] Cohen K, Emmanuel R, Kisin-Finfer E, Shabat D, Peer D (2014). Modulation of drug resistance in ovarian adenocarcinoma using chemotherapy entrapped in hyaluronan-grafted nanoparticle clusters. Acs Nano.

[9] Yamada Y, Hashida M, Harashima H (2015). Hyaluronic acid controls the uptake pathway and intracellular trafficking of an octaarginine-modified gene vector in CD44 positive- and CD44 negative-cells. Biomaterials.

[10] Yang X, Lyer AK, Singh A, Choy E, Hornicek FJ, Amiji MM, Duan Z (2015). MDR1 siRNA loaded hyaluronic acid-based CD44 targeted nanoparticle systems circumvent paclitaxel resistance in ovarian cancer. Scientific Reports.

[11] Liu Y, Ran R, Chen J, Kuang Q, Tang J, Mei L, Zhang Q, Gao H, Zhang Z, He Q (2014). Paclitaxel loaded liposomes decorated with a multifunctional tandem peptide for glioma targeting. Biomaterials.

[12] Avraamides CJ, Garmysusini B, Varner JA (2008). Integrins in angiogenesis and lymphangiogenesis. Nature Reviews Cancer.

[13] Sakurai Y, Hatakeyama H, Sato Y, Hyodo M, Akita H, Ohga N, Hida K, Harashima H (2014). RNAi-mediated gene knockdown and anti-angiogenic therapy of RCCs using a cyclic RGD-modified liposomal-siRNA system. Journal of Controlled Release.

[14] Müller K, Faeh C, Diederich F (2007). Fluorine in pharmaceuticals: looking beyond intuition. Science.

[15] Buer BC, Meagher JL, Stuckey JA, Marsh EN (2012). Structural basis for the enhanced stability of highly fluorinated proteins. Proceedings of the National Academy of Sciences.

[16] Xiong SD, Li L, Jiang J, Tong LP, Wu S, Xu ZS, Chu PK (2010). Cationic fluorine-containing amphiphilic graft copolymers as DNA carriers. Biomaterials.

[17] Wang M, Liu H, Li L, Cheng Y (2014). A fluorinated dendrimer achieves excellent gene transfection efficacy at extremely low nitrogen to phosphorus ratios. Nature Communications.

[18] Ling L, Song L, Liu X, Xi Y, Xia L, Tao H, Ning W, Yang S, Yu C, Tao Y (2017). Artificial Virus Delivers CRISPR-Cas9 System for Genome Editing of Cells in Mice. Acs Nano.

[19] Li L, Song L, Yang X, Li X, Wu Y, He T, Wang N, Yang S, Zeng Y, Yang L (2016). Multifunctional “core-shell” nanoparticles-based gene delivery for treatment of aggressive melanoma. Biomaterials.

[20] He ZY, Wei XW, Luo M, Luo ST, Yang Y, Yu YY, Chen Y, Ma CC, Liang X, Guo FC (2013). Folate-linked lipoplexes for short hairpin RNA targeting claudin-3 delivery in ovarian cancer xenografts. J Control Release.

[21] Bishop CJ, Majewski RL, Guiriba TM, Wilson DR, Bhise NS, Quiñones-Hinojosa A, Green JJ (2016). Quantification of Cellular and Nuclear Uptake Rates of Polymeric Gene Delivery Nanoparticles and DNA Plasmids via Flow Cytometry. Acta Biomaterialia.

